# Cognitive impairment in Chinese patients with isolated generalized dystonia: a case–control study

**DOI:** 10.3389/fneur.2026.1787339

**Published:** 2026-07-01

**Authors:** Shanglin Li, Yingmai Yang, Lin Wang, Jing Gao, Caiyan Liu, Jun Ma, Xinhua Wan

**Affiliations:** 1 Department of Neurology, The First Hospital of Tsinghua University, School of Clinical Medicine, Tsinghua Medicine, Tsinghua University, Beijing, China; 2 Department of Neurology, Peking Union Medical College Hospital, Peking Union Medical College and Chinese Academy of Medical Sciences, Beijing, China; 3 Department of Geriatric Medicine, Qilu Hospital of Shandong University, Jinan, Shandong, China

**Keywords:** Chinese patients, cognition, generalized dystonia, movement disorders, non-motor symptoms

## Abstract

**Background:**

Previous studies have indicated that non-motor symptoms are primary problems in focal dystonia, but limited data are available about cognitive function and their correlation with motor severity in generalized dystonia (GD).

**Methods:**

In the present study, we performed a case–control study and enrolled isolated genetic or idiopathic GD patients and age-, sex- and education-matched healthy controls (HC). Clinical characteristics, motor symptoms, psychiatric symptoms, and cognitive performance were assessed in both groups using various standardized rating scales and a comprehensive neuropsychological battery. Group comparisons, multiple linear regression analyses, and correlation analyses were performed, with adjustment for demographic and affective variables and correction for multiple comparisons.

**Results:**

Twenty patients with GD and matched healthy controls were enrolled and completed the assessments. Compared with HC, GD patients showed mild impairments, particularly in MoCA, executive function/attention, spatial ability, some tests in memory and similarities while other cognitive function did not differ between patients and HC. After adjustment and multiple comparison correction, MOCA, MOCA, executive function/attention and episodic memory remained the most consistent deficits. Several other cognitive differences were no longer significant after correction. No significant associations were found between cognitive performance and motor severity or disease duration. Cognitive performance did not differ between genetic and idiopathic subgroups or between medication groups.

**Conclusion:**

GD is associated with mild and heterogeneous cognitive impairments, with only a subset of deficits remaining robust after adjustment. Cognitive performance appears largely independent of motor severity, disease duration, medication use, and disease etiology.

## Introduction

Dystonia is a movement disorder characterized by sustained or intermittent abnormal movements, postures, or both (1). Although predominantly defined as a motor disorder, non-motor symptoms (NMS) are increasingly recognized as important contributors to the disorder phenotype and disease burden (2). Among these, cognitive dysfunction has emerged as an increasingly important yet incompletely understood component of the dystonia (3–6). Previous studies in focal or segmental dystonia have reported mild deficits in executive function, attention, processing speed, visuospatial abilities, and social cognition (6–8). However, findings regarding the relationship between cognitive dysfunction and disease severity or duration remain inconsistent (3, 9–11), suggesting that cognitive dysfunction may represent an intrinsic non-motor feature of dystonia rather than merely a secondary consequence of motor impairment. Current evidence supports the concept of dystonia as a network disorder involving cortico-basal-ganglia-thalamo-cortical and cerebello–thalamo–cortical circuits (12–14). Structural and functional neuroimaging studies have demonstrated abnormalities not only in basal ganglia but also in frontal, parietal, and cerebellar regions, indicating that widespread network dysfunction may contribute to both motor and non-motor manifestations of dystonia (14). As generalized dystonia (GD) is thought to involve more extensive network abnormalities (15), investigating cognitive function in GD may provide deeper insights into the underlying mechanisms of dystonia.

Despite increasing interest in cognitive function in dystonia, research specifically focusing on generalized dystonia remains scarce and no definitive conclusions have been reached. Previous studies have suggested mild impairment in executive function, attention, verbal fluency, and verbal memory in patients with generalized dystonia (10, 16–18). However, most studies included mixed dystonia subtypes, particularly focal dystonia, while studies specifically focusing on generalized dystonia remain scarce. In several reports, the number of patients with generalized dystonia was extremely small (16, 17), making it difficult to determine whether the reported cognitive abnormalities truly reflect the cognitive profile of generalized dystonia. In addition, whether such subtle cognitive deficits constitute an intrinsic feature of the disease requires further investigation, especially in Chinese generalized dystonia. Therefore, further studies employing comprehensive neuropsychological assessments are needed to clarify the nature and clinical relevance of cognitive dysfunction in generalized dystonia. Our present study aims to investigate cognitive function in Chinese patients with generalized dystonia and to explore its relationship with motor and psychiatric symptoms.

## Materials and methods

### Participants

This single-center case–control (cross-sectional) study included a total of 20 consecutive patients referred to the Department of Neurology in Peking Union Medical College Hospital. The inclusion criteria were inherited or idiopathic GD fulfilling the international standard criteria (a) as determined by a movement disorder specialist, combined with gene testing (targeted gene capture sequencing designed for movement disorders including 148 genes or whole exome sequencing), neuroimages, and laboratory test results, (b) could fully understand the rating scales, and (c) agreed to multiple assessment of cognitive function, (d) ≥ 16 years of age. The exclusion criteria were as follows: (a) clinical or paraclinical findings suggestive of an acquired cause of dystonia, (b) use of antipsychotic drugs or a history of psychosis, (c) other relevant neurological disorders or general comorbidity, and (d) past history of intellectual disability or cognitive impairment. We recruited 20 age- and sex- and education-matched healthy controls (HC) among the healthy residents. None of the controls have any biological relationship with the patients or any clinical symptoms of disease or family history.

### Ethics

This study was approved by the Institutional Review Board and Ethics Committee at Peking Union Medical College Hospital and was performed in accordance with the ethical standards laid down in the 1964 Declaration of Helsinki and its later amendments (approval number: B256). Informed consent was obtained from all participants or their legal guardians.

### Basic clinical characteristics and motor symptoms

Demographic information, including age, sex and education level was obtained from both patients with generalized dystonia (GD) and healthy controls (HC). Additional clinical information, such as disease duration, past history, and family history, was obtained from the patients during an interview conducted by neurologists. All dystonia patients underwent brain magnetic resonance imaging. Motor severity was evaluated according to a standardized videotaped neurological examination based on the Burke-Fahn–Marsden Dystonia Rating Scale motor subscales (BFMDRS-M) (19). The final motor score was determined as the average rating provided by two independent movement disorders specialists.

### Cognitive function evaluation

All participants underwent a series of cognitive tests including Mini-Mental State Examination (MMSE) (20), Montreal Cognitive Assessment (MOCA) (21), Frontal Assessment Battery (FAB) (22), and a battery of comprehensive combined cognitive assessment of executive function/attention (Trail Making Test Part A (23), Symbol Digit Modalities Test (24), the Clock Drawing Test (25), Categorical Verbal Fluency (26), Digit Span Forward (27, 28), Serial Action Imitation), Visuospatial Ability (Copying Test (29), The Benson Complex Figure Test Copy (30), Block Design Test (27, 28), Single Action Imitation),language (Number of Repetition Errors (29), Number of Command-Following Errors, Color Naming, Object Naming), Memory (Associative Learning (31), Episodic Memory (27, 28), Benson Complex Figure Test Recall (32),),and Conceptual Reasoning/Calculation [Similarity Test (27, 28), Arithmetic Calculation (27, 28)]. All participants underwent the Hamilton Anxiety Rating Scale (HAMA) (33), and the Beck Depression Inventory (BDI) (34). All the scales of evaluation were validated Chinese versions.

### Statistical analysis

Statistical analyses were performed using SPSS version 20.0 (SPSS Inc., Chicago, IL, USA) and difference were considered as significant at a two-side *p* < 0.05. Normally distributed data with equal variance were expressed as means ± SD and were compared using independent two-sample t-tests (t), and non-normally distributed data were expressed as median with interquartile range (IQR) and were compared using Mann–Whitney *U*-test. Chi-square test or Fisher’s exact test is used for comparing categorical data between groups. For multiple comparisons, a conservative Bonferroni correction was applied to minimize the risk of type I error. Given the limited and non-systematic missing data as well as the small sample size, analyses were handled using Available-Case (listwise deletion) methods. We also performed Multiple Imputation as a sensitivity analysis, demonstrating the results were fully consistent with those obtained from the Available-Case (listwise deletion) analysis. Multiple linear regression models were used to examine whether cognitive differences between groups remained significant after adjustment for potential confounders, including age, sex, education, HAMA and BDI. Variance inflation factors (VIFs) were used to check for potential multicollinearity among the included covariates. Correlation analysis between variables is performed using Pearson correlation (for normally distributed data) or Spearman correlation (for non-normally distributed data).

## Results

### Demographic and clinical characteristics

According to the inclusion and exclusion criteria, a total of 20 GD patients [mean age 29.55 ± 8.45 years, 9 females (45%)] and 20 age-, sex-, and education-matched healthy controls (HC) [mean age 29.55 ± 9.86 years, 9 females (45%)] completed assessments, including the systematic scales (MMSE, MoCA, and FAB) and a battery of Comprehensive Cognitive Scale assessment. The mean education duration in GD is 12.45 ± 3.41 years with 12.35 ± 2.78 years in HC. The mean BFMDRS-Motor score was 45.7 ± 18.71 points. The etiological distribution included 6 patients with DYT1 (TOR1A gene mutation), 3 with DYT6 (THAP1 gene mutation), and 11 with idiopathic GD. The scores of HAMA and BDI in the GD group were significantly higher than those in the healthy control group, but there were no significant differences in age, sex, or educational level between the two groups (Table 1).

**Table 1:** Demographic information and clinical characteristics in GD and HC.

Variables	GD (*n* = 20)	HC (*n* = 20)	*p*
Age (years)	29.55 ± 8.45	29.55 ± 9.86	0.90
Sex (female)	45%	45%	1.00
Education duration (years)	12.45 ± 3.41	12.35 ± 2.78	0.256
Disease duration (years)	12.25 ± 8.37	-	-
BFMDRS-M	45.7 ± 18.71	-	-
HAMA	10.35 ± 5.21	5.35 ± 4.17	0.002*
BDI	6.5(3–11.75)	2(0.25–6.75)	0.007*

BDI, the Beck Depression Inventory; BFMDRS-M, Burke–Fahn–Marsden dystonia rating scale motor subscales; GD, generalized dystonia; HAMA, Hamilton Anxiety Rating Scale; HC, healthy controls; *P < 0.05.

### Global cognitive function

The median (interquartile range [IQR]) score of the Mini-Mental State Examination (MMSE) in patients was 29.00 (28.00–30.00), showing no significant difference from that in healthy controls (median [IQR]: 30.00 [28.25–30.00]). In contrast, the Montreal Cognitive Assessment (MoCA) and Frontal Assessment Battery (FAB) scores in the patient group were 25.65 ± 2.64 (mean ± standard deviation) and 16.50 (15.00–17.75) (median [IQR]), respectively. These scores were significantly lower than those in the control group, which were 28.60 ± 0.88 (mean ± standard deviation) for MoCA and 18.00 (17.25–18.00) (median [IQR]) for FAB, with statistically significant differences (*p* < 0.001). (Table 2).

**Table 2:** Global cognitive function of GD and HC.

Variables	GD (*n* = 20)	HC (*n* = 20)	*p*
MMSE	29.00(28.00–30.00)	30.00(28.25–30.00)	0.438
MoCA	25.65 ± 2.64	28.6 ± 0.88	0.000*
FAB	16.50(15.00–17.75)	18.00(17.25–18.00)	0.000*

MMSE, Mini-Mental State Examination; MOCA, Montreal Cognitive Assessment; FAB, Frontal Assessment Battery; GD, generalized dystonia; HC, healthy controls; *P < 0.05.

### Performance in different cognitive domains

Performance across cognitive domains is summarized in Table 3. Multiple comparisons were corrected using the Bonferroni method (adjusted significance threshold *p* < 0.00238). All *p*-values reported are unadjusted.

**Table 3:** Performance in different cognitive domains of GD and HC.

Domain	Test	GD		HC		*P*
		Value	*n*	Value	*n*	
Executive function/attention	CVF	20.53 ± 5.79	17	23.35 ± 5.64	20	0.438
	SDMT	39.25 ± 11.72	20	67.00 ± 11.28	20	0.000*
	CDT	3(2.25–4)	20	4(4–4)	20	0.005
	TMTA(s)	53.39 ± 19.39	20	41.32 ± 16.98	20	0.043
	DSF	8(8–9)	19	9(8–10)	20	0.107
	Serial Action Imitation	2(1.25–3)	20	3(3,3)	20	0.000*
Visuospatial ability	Copying Test	10(8.25–10)	20	10(10–10)	20	0.050
	BCFT-C	16(15–16)	20	16.5(16–17)	20	0.000*
	BDT	9(7.31–9)	20	9(9–9)	20	0.657
	Single Action Imitation	7(7–7)	20	7(7–7)	20	0.877
Language	Object naming	10(10–10)	20	10(10–10)	20	0.075
	Color naming	6(6–6)	20	6(6–6)	20	0.317
	Repetition Errors	0(0–2)	20	0(0–0)	20	0.010
	Command-Following Errors	0(0–1)	20	0(0–0)	20	0.004
Memory	Associative Learning	11.68 ± 4.45	20	15.45 ± 6.85	20	0.046
	Episodic Memory	8.55 ± 3.62	20	14.32 ± 2.66	20	0.000*
	BCFT-R	13(12–16)	20	15(13.25–16.75)	20	0.090
Conceptual reasoning/Calculation	Similarity Test	15.9 ± 4.19	20	20.20 ± 2.09	20	0.000*
	Arithmetic Calculation	10.8 ± 3.98	20	13.55 ± 3.39	20	0.024

Data are presented as mean ± standard deviation (SD) or median (interquartile range, IQR) according to data distribution. CVF, Categorical Verbal Fluency; SDMT, Symbol Digit Modalities Test; CDT, Clock Drawing Test; TMTA, Trail Making Test part A; DSF, Digit Span Forward; BCFT-C, Benson Complex Figure Test–Copy; BCFT-R, Benson Complex Figure Test–Recall; BDT, Block Design Test; GD, generalized dystonia; HC, healthy controls. Multiple comparisons were corrected using Bonferroni adjustment. * Means remained significant after Bonferroni correction (adjusted threshold p<0.00238 ).

#### Executive function/attention

In the domain of executive function/attention, GD patients performed significantly worse than HC in the Symbol Digit Modalities Test (SDMT) and Serial Action Imitation, and these differences remained significant after correction for multiple comparisons (*p* < 0.0001). Clock Drawing Test (CDT) and Trail Making Test Part A (TMTA) showed group differences at the uncorrected level (*p* < 0.05), but these differences did not survive Bonferroni correction. No differences were observed in their performance in Category Verbal Fluency and Forward Digit Span between two groups.

#### Visuospatial ability

In the visuospatial domain, GD patients exhibited significantly lower performance than healthy controls on the Benson Complex Figure Test-Copy (*p* < 0.0001). Total scores of copying, single action imitation, and block design tests were comparable between the two groups.

#### Language

When it comes to language function, No significant differences were observed between groups in object naming or color naming. GD patients tended showed a higher number of errors in repetition and command-following tasks compared with healthy controls (both *p* < 0.05), but these differences did not survive Bonferroni correction.

#### Memory

In the field of memory, GD patients demonstrated significantly lower performance in episodic memory scores compared with healthy controls (*p* < 0.0001). They also demonstrated significantly poorer performance in associative learning compared with controls, but this did not survive correction. No difference was found between the two groups in BCFT recall.

#### Conceptual reasoning/calculation

In the conceptual reasoning domain, GD patients performed worse in Similarity Test (*p* < 0.0001) compared with healthy controls. Although Arithmetic Calculation scores were lower in GD patients, the difference did not remain significant after Bonferroni correction.

### Further analysis

#### Multiple linear regression analysis

Since age, sex, education level, anxiety, and depression may affect cognitive function, although we had selected age-, sex-, and education-matched normal controls to minimize the impact of these factors, multiple linear regression analysis was further applied for adjustment to clarify the correlation with the disease more precisely. After adjusting for age, sex, education level, Beck Depression Inventory (BDI), and Hamilton Anxiety Rating Scale (HAMA), the Montreal Cognitive Assessment (MoCA), Symbol Digit Modalities Test (SDMT), and Episodic Memory were still significantly correlated with GD, and these correlations remained significant after correction for multiple comparisons. Other measures, including TMTA, BCFT-C, FAB, similarity test, and copying showed similar group trends but did not survive Bonferroni correction. Of note, Clock Design Test (CDT) did not reach significance after Bonferroni correction in the unadjusted analysis but showed a significant association in the adjusted regression model. Detailed results are provided in Table 4. All VIFs were below commonly accepted thresholds, suggesting that multicollinearity was unlikely to have influenced the regression results.

**Table 4:** Correlations between cognitive function and motor severity

Variables	BFMDRS-M		Disease duration	
	ρ	*p*	ρ	*p*
MMSE	0.06	0.797	–0.23	0.330
MoCA	–0.50	0.023	–0.09	0.700
FAB	–0.14	0.567	–0.03	0.884
CVF	0.00	0.999	–0.30	0.240
SDMT	–0.60	0.005	–0.15	0.521
CDT	0.11	0.634	0.24	0.307
TMTA(s)	0.53	0.017	0.29	0.221
DSF	0.00	0.985	0.11	0.657
Verbal command following errors	0.21	0.379	0.01	0.958
Copying test	–0.12	0.608	0.06	0.816
BCFT-C	–0.51	0.020	0.05	0.845
BCFT-R	–0.36	0.118	0.27	0.246
BDT	–0.25	0.288	0.01	0.975
Object naming	–0.22	0.354	0.10	0.681
Color naming	–0.10	0.677	–0.28	0.230
Repetition errors	0.09	0.721	–0.04	0.865
Associative learning	–0.10	0.665	–0.08	0.730
Episodic memory	–0.32	0.169	–0.22	0.361
Similarity test	–0.11	0.640	–0.32	0.172
Arithmetic calculation	–0.05	0.836	–0.06	0.817
Single action imitation	–0.27	0.241	0.14	0.553
Serial action imitation	0.15	0.521	–0.03	0.911

CVF, Categorical Verbal Fluency; SDMT, Symbol Digit Modalities Test; CDT, Clock Drawing Test; TMTA, Trail Making Test part A; DSF, Digit Span Forward; BCFT-C, Benson Complex Figure Test–Copy; BCFT-R, Benson Complex Figure Test–Recall; BDT, Block Design Test; GD, generalized dystonia; HC, healthy controls.

#### Correlation analysis

To clarify the relationship of cognitive function and motor severity, further correlation analysis was conducted between each cognitive function and the Burke-Fahn-Marsden Dystonia Rating Scale-Motor (BFMDRS-M) score as well as disease duration. Although some measures, including MoCA, SDMT, TMTA, and the Benson Complex Figure Test Copy, initially showed nominal associations with motor severity (*p* < 0.05), none of these correlations survived Bonferroni correction. No significant correlations were observed between cognitive measures and disease duration. Detailed results are provided in Table 5.

**Table 5:** Multiple linear regression of cognitive performance in GD and HC.

Test	B (95% CI)	β (Std.)	p	Adj. p
MMSE	0.47 (–0.50, 1.44)	0.17	0.332	≥0.05
MOCA	2.98 (1.61 4.36)	0.62	<0.001	0.002*
FAB	1.16 (0.32, 2.00)	0.40	0.008	≥0.05
CVF	3.15 (–0.54, 6.83)	0.28	0.091	≥0.05
SDMT	25.94 (18.07, 33.81)	0.73	<0.001	<0.001*
CDT	0.89 (0.38, 1.41)	0.53	0.001	0.022*
TMTA(s)	–13.29 (–24.54, –2.04)	–0.35	0.022	≥0.05
DSF	0.73 (–0.10,1.57)	0.32	0.084	≥0.05
Command following errors	–0.46 (–1.07, 0.15)	–0.26	0.133	≥0.05
Copying test	0.89 (0.09, 1.69)	0.40	0.030	≥0.05
BCFT-C	1.27 (0.34, 2.21)	0.47	0.009	≥0.05
BCFT-R	1.17 (–0.57, 2.90)	0.23	0.180	≥0.05
BDT	0.93 (–0.35, 2.20)	0.26	0.148	≥0.05
Object naming	0.16 (–0.02, 0.34)	0.31	0.075	≥0.05
Color naming	0.10 (–0.13, 0.33)	0.16	0.391	≥0.05
Repetition errors	–1.46 (–3.41,0.49)	–0.26	0.136	≥0.05
Associative learning	2.91 (–0.62, 6.45)	0.25	0.103	≥0.05
Episodic memory	4.66 (2.44, 6.89)	0.55	<0.001	0.003*
Similarity test	3.25 (1.04, 5.45)	0.42	0.005	≥0.05
Arithmetic calculation	2.38 (–0.15, 4.91)	0.31	0.065	≥0.05
Single action imitation	0.15 (–0.27, 0.58)	0.14	0.467	≥0.05
Serial action imitation	0.60 (–0.05, 1.25)	0.34	0.069	≥0.05

CVF, Categorical Verbal Fluency; SDMT, Symbol Digit Modalities Test; CDT, Clock Drawing Test; TMTA, Trail Making Test part A; DSF, Digit Span Forward; BCFT-C, Benson Complex Figure Test–Copy; BCFT-R, Benson Complex Figure Test–Recall; BDT, Block Design Test; GD, generalized dystonia; HC, healthy controls. Adj. *p* = Bonferroni‑corrected *p-*value, *P < 0.05.

#### Subgroup analysis

##### Impact of medications on cognitive function

During the treatment of GD, patients may receive medications that exert a negative impact on cognitive function, such as trihexyphenidyl and benzodiazepines. Patients were divided according to the use of medications that may affect cognition (e.g., anticholinergics and benzodiazepines). The results showed no significant differences in all cognitive functions between the two groups (see Table 6). This suggests that medications did not cause significant cognitive function differences in GD patients in this study.

**Table 6:** Cognitive function in GD patients with and without medication.

Variables		GD with medicine(*n* = 11)	GD without medicine (*n* = 9)	*p*
Global Cognition	MMSE	29 (28-30)	29 (28-30)	0.552
	MoCA	25.45 ± 2.42	25.89 ± 3.02	0.725
	FAB	16 (15-17)	17 (16-18)	0.703
Executive function/Attention	CVF	21.13 ± 6.1	20 ± 5.81	0.703
	SDMT	35.64 ± 11.66	43.67 ± 10.78	0.819
	CDT	4 (3-4)	2 (2-3)	0.094
	TMTA(s)	59.66 ± 24.03	45.73 ± 7.16	0.281
	DSF	9 (8-9)	8 (7-9)	0.243
	Serial Action Imitation	2 (1-3)	2 (2-2)	0.525
Visuospatial ability	Copying Test	10 (9–10)	9 (8–10)	0.095
	BCFT-C	16 (15–16)	16 (15–16)	0.536
	BDT	9 (9–9)	9 (6.75–9)	0.658
	Single Action Imitation	7 (7–7)	7 (7–7)	1.000
Language	Object naming	10 (10-10)	10 (10–10)	0.471
	Color naming	6 (6–6)	6 (6–6)	0.328
	Repetition errors	0 (0–9)	0 (0–2)	0.900
	Command following errors	0 (0–1)	0 (0–1)	1.000
Memory	Associative learning	10.77 ± 4.78	12.78 ± 4.01	0.910
	Episodic memory	7.59 ± 3.25	9.72 ± 3.89	0.312
	BCFT-R	14 (12–14)	13 (12–16)	0.848
Conceptual reasoning/calculation	Similarity test	16.00 ± 4.12	15.78 ± 4.52	0.845
	Arithmetic calculation	11.64 ± 2.80	9.78 ± 5.07	0.775

Data are presented as mean ± standard deviation (SD) or median (interquartile range, IQR) according to data distribution. CVF, Categorical Verbal Fluency; SDMT, Symbol Digit Modalities Test; CDT, Clock Drawing Test; TMTA, Trail Making Test part A; DSF, Digit Span Forward; BCFT-C, Benson Complex Figure Test–Copy; BCFT-R, Benson Complex Figure Test–Recall; BDT, Block Design Test; GD, generalized dystonia; HC, healthy controls. Multiple comparisons were corrected using Bonferroni adjustment. * Means remained significant after Bonferroni correction (adjusted threshold p<0.00238 ).

##### Impact of etiology on cognitive function

Patients with generalized dystonia were further divided into genetic and idiopathic subgroups. No significant differences were observed in any of the cognitive measures between the two groups (see Table 7 for details).

**Table 7:** Cognitive function in genetic and idiopathic generalized dystonia.

Domain	Test	Hereditary GD	Idiopathic GD	p
Global cognition	MMSE	30 (29-30)	29(28-30)	0.430
	MoCA	25.89±2.76	25.65 ± 2.66	0.725
	FAB	17 (16-17)	16(14-18)	0.384
Executive function/attention	CVF	22.43±8.68	19.20 ± 2.15	0.942
	SDMT	43.22±14.58	36.00 ± 8.06	0.124
	CDT	3 (2-4)	3 (3-4)	0.780
	TMTA(s)	48.49±22.05	57.40 ± 16.91	0.281
	DSF	9 (8-9)	8 (8-9)	0.828
	Serial action imitation	2 (2-3)	2 (1-3)	0.873
Visuospatial ability	Copying Test	10 (8-10)	10 (9-10)	0.804
	BCFT-C	16 (15-16)	16 (15-16)	1.000
	BDT	9 (9-9)	9 (6.75-9)	0.265
	Single action imitation	7 (7-7)	7 (6-7)	0.072
Language	Object naming	10 (10-10)	10 (10-10)	0.717
	Color naming	6 (6-6)	6 (6-6)	0.328
	Repetition errors	0 (0-0)	1 (0-9)	0.172
	Command following errors	0 (0-1)	0 (0-1)	1.000
Memory	Associative learning	11.94 ± 4.23	11.45 ± 4.82	0.926
	Episodic memory	9.44 ± 3.23	7.82 ± 3.91	0.602
	BCFT-R	14 (12-16)	13 (11–16)	0.274
Conceptual reasoning/calculation	Similarity test	16 ± 5.74	15.82 ± 2.64	0.554
	Arithmetic calculation	11.33 ± 3.08	10.36 ± 4.7	0.262

Data are presented as mean ± standard deviation (SD) or median (interquartile range, IQR) according to data distribution. CVF, Categorical Verbal Fluency; SDMT, Symbol Digit Modalities Test; CDT, Clock Drawing Test; TMTA, Trail Making Test part A; DSF, Digit Span Forward; BCFT-C, Benson Complex Figure Test–Copy; BCFT-R, Benson Complex Figure Test–Recall; BDT, Block Design Test; GD, generalized dystonia; HC, healthy controls. Multiple comparisons were corrected using Bonferroni adjustment. * Means remained significant after Bonferroni correction (adjusted threshold p<0.00238 ).

## Conclusion

Compared with HC, GD patients showed mild cognitive impairments, mainly in MoCA, executive function/attention, visuospatial ability, some tests in memory and similarities while other cognitive function was relatively preserved. After adjustment for demographic and psychiatric factors and correction for multiple comparisons, MOCA, executive function/attention and episodic memory remained significantly affected, suggesting a more stable cognitive component. Some of the cognitive differences were no longer significant after adjustment, indicating that these may be influenced by demographic and psychiatric factors. Cognitive performance appears largely independent of motor severity, disease duration, medication use, and disease etiology.

## Discussion

Cognitive impairment is not traditionally regarded as a core feature of dystonia progression, and this was also reflected in our study. No significant difference in MMSE scores was observed between patients with generalized dystonia (GD) and healthy controls, suggesting relatively preserved global cognitive function at a screening level. However, GD patients showed significantly lower MOCA scores even after Bonferroni correction, indicating that subtle cognitive dysfunction may be detected using more sensitive measures. These findings are consistent with previous studies in idiopathic and DYT1-related dystonia showing largely preserved global cognition despite subtle deficits (16, 17, 35, 36), and are further supported by studies in focal dystonia demonstrating reduced MOCA performances (37).

Our findings suggest that cognitive impairment in isolated generalized dystonia (GD) may follow a domain-specific pattern rather than reflecting global decline. In our study, GD patients performed worse mainly on the Frontal Assessment Battery (FAB), Symbol Digit Modalities Test (SDMT), Episodic Memory, Serial Action Imitation, and Benson Complex Figure Test-Copy (BCFT-C), and Similarity Test after Bonferroni correction, whereas language- and calculation-related functions were relatively preserved. These findings suggest that cognitive deficits in patients with GD primarily involve executive function, attention, and visuospatial processing, which is in line with previous studies (16, 17). In addition, poorer performance on episodic memory and the Similarity Test points to milder involvement of memory and abstract reasoning abilities. Patients with GD also showed significantly lower FAB scores compared with healthy controls, supporting the presence of frontal lobe-related dysfunction. Moreover, the decline in executive and attentional functions may also contribute to the reduced performance in MoCA (given that the Clock Drawing Test is also an item included in MoCA). All these results imply that frontal lobe dysfunction may exist in GD patients. In contrast, Categorical Verbal Fluency (CVF) did not differ between GD patients and healthy controls in the present study, suggesting that this aspect of language-related executive function may be relatively preserved in GD. This finding differs from some previous studies reporting CVF impairments in dystonia (16, 17). However, there are inconsistencies among different research findings: some studies reported that dystonia patients performed worse than healthy controls (16) whereas others found no significant differences (6). Therefore, the current results should be interpreted cautiously considering the cross-sectional study design and relatively small sample size.

As is well known, cognitive function may be influenced by several factors, including age, sex, education level and psychopathological symptoms, especially depression. Previous studies and our own findings have proved psychopathological symptoms in GD patients (38). Thus, it remains unclear whether cognitive impairment constitutes a primary manifestation of disease itself or is partly driven by these confounding factors. To further explore this issue, we performed multiple linear regression analyses adjusting for age, sex, education, anxiety, and depression. After adjustment and multiple comparisons, the most consistent cognitive differences between patients with GD and healthy controls were observed in MOCA, SDMT, and episodic memory. CDT also showed a significant effect in the adjusted model. Overall, these findings suggest that cognitive changes in GD may reflect a mild and relatively distributed reduction in cognitive efficiency, mainly involving executive function/attention, visuospatial processing and episodic memory. In contrast, after adjusting for age, sex, education, anxiety, depression, and correcting for multiple comparisons, Serial Action Imitation, BCFT copy, FAB, and Similarity Test were no longer significant. This pattern suggests that part of the variability in cognitive performance may be related to demographic and emotional factors, as well as the fact that some cognitive tests are influenced by multiple underlying cognitive processes. Previous studies have also generally reported that cognitive changes in dystonia are mild and heterogeneous, with only weak and inconsistent relationships with mood symptoms, while one study reported small correlations between BDI scores and certain executive function tasks (36). Taken together, our findings support a mixed profile, where some cognitive changes may reflect more stable disease-related features, while others are likely influenced by affective states and methodological factors. Given the limited sample size and exploratory nature of multiple testing, these results should be interpreted cautiously.

Dystonia is a movement disorder characterized by sustained or intermittent muscle contractions causing abnormal repetitive movements. Therefore, it remains of interest whether the observed cognitive changes are secondary to motor symptoms or reflect more independent disease-related processes. Previous studies in focal dystonia have reported inconsistent findings regarding the relationship between motor severity and cognitive function. For example, in a study of blepharospasm, botulinum toxin injection improved motor symptoms and patients’ performance on sustained attention tests (39). Conversely, other studies have found no association between the severity or duration of blepharospasm symptoms and cognitive function (3, 4). In our study, correlation analysis was conducted to examine the relationships between cognitive function and motor symptom severity (assessed by the Burke-Fahn-Marsden Dystonia Rating Scale-Motor, BFMDRS-M) as well as disease duration in GD patients. After correction for multiple comparisons, no significant correlations were found between any cognitive measure and either motor symptom severity or disease duration. In conclusion, these results do not support a robust relationship between cognitive performance and motor severity or disease duration in GD. This dissociation between motor severity and cognitive performance is also supported by neuroimaging evidence in DYT1 mutation carriers, in whom similar deficits in motor sequence learning and visuospatial processing have been observed regardless of whether clinical motor symptoms are present (40, 41). These findings further suggest that certain cognitive abnormalities in dystonia may not be directly dependent on overt motor manifestations. However, given the cross-sectional design of this study, causal relationships cannot be inferred. Future longitudinal studies with larger samples are needed to further clarify the relationship between motor and cognitive features in dystonia and their underlying pathophysiological mechanisms.

Patients with dystonia often receive anticholinergic drugs or benzodiazepines, both of which may potentially affect cognitive performance. To address this potential confounding factor, subgroup analysis was conducted to examine whether medication use influenced cognitive outcomes in GD patients. The results showed no significant differences in any cognitive measures between patients receiving medication and those who were not. These findings suggest that the observed cognitive impairments in GD are unlikely to be primarily driven by medication status in our sample. However, this interpretation should be made with caution, given the heterogeneity of medication types, doses, and treatment duration. To further explore whether disease heterogeneity might account for cognitive differences, patients with GD were also stratified into genetic and idiopathic subgroups. No significant differences were observed between the two groups across cognitive measures, suggesting that etiology, at least at this level of classification, does not appear to have a measurable impact on cognitive outcomes in our study. However, previous studies have reported cognitive and developmental impairments in patients with DYT28 (KMT2B-related dystonia), which can present as generalized dystonia (42, 43), indicating that cognitive features may differ across genetic subtypes. Due to the limited sample size, more detailed genetic stratification was not feasible in this study, and future research with larger cohorts is needed to systematically investigate genotype–phenotype associations in cognitive function among dystonia patients.

From a neurobiological perspective, current evidence supports the view that dystonia is not a focal basal ganglia disorder but rather a brain network-based syndrome involving cortico–basal ganglia–thalamo–cortical and cerebello–thalamo–cortical circuits (12–14, 44, 45). Neuroimaging studies have demonstrated abnormal functional changes and connectivity not only within the basal ganglia, but also across the dorsolateral prefrontal cortex (DLPFC), anterior cingulate cortex (ACC), supplementary motor area (SMA), cerebellar regions, and parietal regions, suggesting that dysfunction extends beyond motor execution areas to include higher-order cognitive control networks (46, 47). The cognitive outcomes observed in our study may reflect disruption affecting multiple systems. In particular, impairments in processing speed, attention and global cognitive efficiency (as reflected by SDMT and MoCA) may be associated with altered frontoparietal, anterior cingulate connectivity, and cerebello-thalamo-cortical pathways (12, 14). In addition, episodic memory performance may be influenced by disrupted interactions between cortical and subcortical regions. However, given the absence of neuroimaging data in the present study, these interpretations remain speculative.

This study has several limitations that should be acknowledged. Firstly, the relatively small sample size may have limited the statistical power and generalizability of the findings, although generalized dystonia is a rare disorder and the sample size in our study was comparable to that of previous studies in this field. Future multicenter studies with larger cohorts are needed to further validate these results. Secondly, the study population mainly consisted of young adults, and several comprehensive cognitive scales lack well-established normative data for individuals under 20 years or in the 20–30 age range. As a result, cognitive performance in GD patients was compared with age-, sex-, and education-matched healthy controls rather than population-based normative data. Thirdly, although several potential confounding factors were statistically adjusted for, the influence of motor symptoms on cognitive testing cannot be completely excluded. This is particularly relevant for tasks involving visuomotor coordination, drawing, or writing-related components, such as copying tasks, CDT, and SDMT. In addition, due to the limited sample size, we were unable to further stratify patients according to the anatomical distribution of dystonia symptoms, which may differentially affect cognitive task performance. Fourthly, psychiatric symptoms, especially anxiety and depression, may also influence cognitive performance. Although these factors were adjusted for in the regression analyses, their influence on cognition cannot be completely excluded in a cross-sectional study. In addition, the present study included a genetically heterogeneous cohort (including DYT1, DYT6, and idiopathic GD), which may limit the ability to detect subtype-specific cognitive differences. Finally, cross-sectional design limits conclusions regarding causality and does not allow assessment of the longitudinal course of cognitive dysfunction in GD. As a result, it remains unclear whether the observed cognitive changes are stable or progressive over time. Future longitudinal studies with larger samples and multimodal imaging will be important to better characterize the temporal course and underlying mechanisms of cognitive changes in dystonia.

## Data Availability

The original contributions presented in the study are included in the article/Supplementary material, further inquiries can be directed to the corresponding author/s.
