# Supplementary material for: Cognitive impairment in Chinese patients with isolated generalized dystonia: a case–control study
Source: Front Neurol. 2026 Jul 1;17:1787339. doi: 10.3389/fneur.2026.1787339 (PMC13369596; doi:10.3389/fneur.2026.1787339)
Supplement: Supplementary file 1 [file Supplementary_file_1.DOCX]

**Supplementary Table 1. Correlations between cognitive function and motor severity**

| Variables | BFMDRS-M | | Disease duration | |
| --- | --- | --- | --- | --- |
|  | ρ | p | ρ | p |
| MMSE | 0.06 | 0.797 | -0.23 | 0.330 |
| MoCA | -0.50 | 0.023 | -0.09 | 0.700 |
| FAB | -0.14 | 0.567 | -0.03 | 0.884 |
| CVF | 0.00 | 0.999 | -0.30 | 0.240 |
| SDMT | -0.60 | 0.005 | -0.15 | 0.521 |
| CDT | 0.11 | 0.634 | 0.24 | 0.307 |
| TMTA(s) | 0.53 | 0.017 | 0.29 | 0.221 |
| DSF | 0.00 | 0.985 | 0.11 | 0.657 |
| Verbal command following errors | 0.21 | 0.379 | 0.01 | 0.958 |
| Copying Test | -0.12 | 0.608 | 0.06 | 0.816 |
| BCFT-C | -0.51 | 0.020 | 0.05 | 0.845 |
| BCFT-R | -0.36 | 0.118 | 0.27 | 0.246 |
| BDT | -0.25 | 0.288 | 0.01 | 0.975 |
| Object naming | -0.22 | 0.354 | 0.10 | 0.681 |
| Color naming | -0.10 | 0.677 | -0.28 | 0.230 |
| Repetition Errors | 0.09 | 0.721 | -0.04 | 0.865 |
| Associative Learning | -0.10 | 0.665 | -0.08 | 0.730 |
| Episodic Memory | -0.32 | 0.169 | -0.22 | 0.361 |
| Similarity Test | -0.11 | 0.640 | -0.32 | 0.172 |
| Arithmetic Calculation | -0.05 | 0.836 | -0.06 | 0.817 |
| Single Action Imitation | -0.27 | 0.241 | 0.14 | 0.553 |
| Serial Action Imitation | 0.15 | 0.521 | -0.03 | 0.911 |

Abbreviations: CVF, Categorical Verbal Fluency; SDMT, Symbol Digit Modalities Test; CDT, Clock Drawing Test; TMTA, Trail Making Test part A; DSF, Digit Span Forward; BCFT-C, Benson Complex Figure Test–Copy; BCFT-R, Benson Complex Figure Test–Recall; BDT, Block Design Test; GD, generalized dystonia; HC, healthy controls.

**Supplementary Table 2. Multiple linear regression of cognitive performance in GD and HC**

| Test | | B (95% CI) | β (Std.) | p | Adj. p |
| --- | --- | --- | --- | --- | --- |
| MMSE | | 0.47(-0.50, 1.44) | 0.17 | 0.332 | ≥0.05 |
| MOCA | | 2.98(1.61 4.36) | 0.62 | <0.001 | 0.002* |
| FAB | | 1.16(0.32, 2.00) | 0.40 | 0.008 | ≥0.05 |
| CVF | | 3.15(-0.54, 6.83) | 0.28 | 0.091 | ≥0.05 |
| SDMT | | 25.94(18.07, 33.81) | 0.73 | <0.001 | <0.001* |
| CDT | | 0.89(0.38, 1.41) | 0.53 | 0.001 | 0.022* |
| TMTA(s) | | -13.29(-24.54, -2.04) | -0.35 | 0.022 | ≥0.05 |
| DSF | | 0.73(-0.10,1.57) | 0.32 | 0.084 | ≥0.05 |
| Command Following Errors | | -0.46(-1.07, 0.15) | -0.26 | 0.133 | ≥0.05 |
| Copying Test | | 0.89(0.09, 1.69) | 0.40 | 0.030 | ≥0.05 |
| BCFT-C | | 1.27(0.34, 2.21) | 0.47 | 0.009 | ≥0.05 |
| BCFT-R | 1.17(-0.57, 2.90) | | 0.23 | 0.180 | ≥0.05 |
| BDT | 0.93(-0.35, 2.20) | | 0.26 | 0.148 | ≥0.05 |
| Object naming | 0.16 (-0.02, 0.34) | | 0.31 | 0.075 | ≥0.05 |
| Color naming | 0.10(-0.13, 0.33) | | 0.16 | 0.391 | ≥0.05 |
| Repetition Errors | -1.46 (-3.41,0.49) | | -0.26 | 0.136 | ≥0.05 |
| Associative Learning | 2.91 (-0.62, 6.45) | | 0.25 | 0.103 | ≥0.05 |
| Episodic Memory | 4.66 (2.44, 6.89) | | 0.55 | <0.001 | 0.003* |
| Similarity Test | 3.25(1.04, 5.45) | | 0.42 | 0.005 | ≥0.05 |
| Arithmetic Calculation | 2.38(-0.15, 4.91) | | 0.31 | 0.065 | ≥0.05 |
| Single Action Imitation | 0.15 (-0.27, 0.58) | | 0.14 | 0.467 | ≥0.05 |
| Serial Action Imitation | 0.60(-0.05, 1.25) | | 0.34 | 0.069 | ≥0.05 |

Note: CVF, Categorical Verbal Fluency; SDMT, Symbol Digit Modalities Test; CDT, Clock Drawing Test; TMTA, Trail Making Test part A; DSF, Digit Span Forward; BCFT-C, Benson Complex Figure Test–Copy; BCFT-R, Benson Complex Figure Test–Recall; BDT, Block Design Test; GD, generalized dystonia; HC, healthy controls. Adj. p = Bonferroni‑corrected p value, * P<0.05

**Supplementary Table 3. Cognitive function in GD patients with and without medication**

| Variables | | GD with medicine（n=11） | GD without medicine  (n=9) | p |
| --- | --- | --- | --- | --- |
| Global Cognition | MMSE | 29(28-30) | 29(28-30) | 0.552 |
|  | MoCA | 25.45±2.42 | 25.89±3.02 | 0.725 |
|  | FAB | 16(15-17) | 17(16-18) | 0.703 |
| Executive Function/ Attention | CVF | 21.13±6.1 | 20±5.81 | 0.703 |
|  | SDMT | 35.64±11.66 | 43.67±10.78 | 0.819 |
|  | CDT | 4 (3-4) | 2(2-3) | 0.094 |
|  | TMTA(s) | 59.66±24.03 | 45.73±7.16 | 0.281 |
|  | DSF | 9(8-9) | 8(7-9) | 0.243 |
|  | Serial Action Imitation | 2(1-3) | 2(2-2) | 0.525 |
| Visuospatial Ability | Copying Test | 10(9-10) | 9(8-10) | 0.095 |
|  | BCFT-C | 16(15-16) | 16(15-16) | 0.536 |
|  | BDT | 9(9-9) | 9(6.75-9) | 0.658 |
|  | Single Action Imitation | 7(7-7) | 7(7-7) | 1.000 |
| Language | Object naming | 10(10-10) | 10(10-10) | 0.471 |
|  | Color naming | 6(6-6) | 6(6-6) | 0.328 |
|  | Repetition Errors | 0(0-9) | 0(0-2) | 0.900 |
|  | Command Following  Errors | 0(0-1) | 0(0-1) | 1.000 |
| Memory | Associative Learning | 10.77±4.78 | 12.78±4.01 | 0.910 |
|  | Episodic Memory | 7.59±3.25 | 9.72±3.89 | 0.312 |
|  | BCFT-R | 14(12-14) | 13(12-16) | 0.848 |
| Conceptual Reasoning  /Calculation | Similarity Test | 16.00±4.12 | 15.78±4.52 | 0.845 |
|  | Arithmetic Calculation | 11.64±2.80 | 9.78±5.07 | 0.775 |

Note: Data are presented as mean ± standard deviation (SD) or median (interquartile range, IQR) according to data distribution. CVF, Categorical Verbal Fluency; SDMT, Symbol Digit Modalities Test; CDT, Clock Drawing Test; TMTA, Trail Making Test part A; DSF, Digit Span Forward; BCFT-C, Benson Complex Figure Test–Copy; BCFT-R, Benson Complex Figure Test–Recall; BDT, Block Design Test; GD, generalized dystonia; HC, healthy controls. Multiple comparisons were corrected using Bonferroni adjustment. *means remained significant after Bonferroni correction (adjusted threshold $p<0.00238$).

**Supplementary Table 4. Cognitive Function in Genetic and Idiopathic Generalized Dystonia**

| Domain | Test | Hereditary GD | Idiopathic GD | p |
| --- | --- | --- | --- | --- |
| Global Cognition | MMSE | 30(29-30) | 29(28-30) | 0.430 |
|  | MoCA | 25.89±2.76 | 25.65±2.66 | 0.725 |
|  | FAB | 17(16-17) | 16(14-18) | 0.384 |
| Executive Function/ Attention | CVF | 22.43±8.68 | 19.20±2.15 | 0.942 |
|  | SDMT | 43.22±14.58 | 36.00±8.06 | 0.124 |
|  | CDT | 3(2-4) | 3(3-4) | 0.780 |
|  | TMTA(s) | 48.49±22.05 | 57.40±16.91 | 0.281 |
|  | DSF | 9(8-9) | 8(8-9) | 0.828 |
|  | Serial Action Imitation | 2(2-3) | 2(1-3) | 0.873 |
| Visuospatial Ability | Copying Test | 10(8-10) | 10(9-10) | 0.804 |
|  | BCFT-C | 16(15-16) | 16(15-16) | 1.000 |
|  | BDT | 9(9-9) | 9(6.75-9) | 0.265 |
|  | Single Action Imitation | 7(7-7) | 7(6-7) | 0.072 |
| Language | Object naming | 10(10-10) | 10(10-10) | 0.717 |
|  | Color naming | 6(6-6) | 6(6-6) | 0.328 |
|  | Repetition Errors | 0（0-0） | 1(0-9) | 0.172 |
|  | Command Following  Errors | 0(0-1) | 0(0-1) | 1.000 |
| Memory | Associative Learning | 11.94±4.23 | 11.45±4.82 | 0.926 |
|  | Episodic Memory | 9.44±3.23 | 7.82±3.91 | 0.602 |
|  | BCFT-R | 14(12-16) | 13(11-16) | 0.274 |
| Conceptual Reasoning  /Calculation | Similarity Test | 16±5.74 | 15.82±2.64 | 0.554 |
|  | Arithmetic Calculation | 11.33±3.08 | 10.36±4.7 | 0.262 |

Note: Data are presented as mean ± standard deviation (SD) or median (interquartile range, IQR) according to data distribution. CVF, Categorical Verbal Fluency; SDMT, Symbol Digit Modalities Test; CDT, Clock Drawing Test; TMTA, Trail Making Test part A; DSF, Digit Span Forward; BCFT-C, Benson Complex Figure Test–Copy; BCFT-R, Benson Complex Figure Test–Recall; BDT, Block Design Test; GD, generalized dystonia; HC, healthy controls. Multiple comparisons were corrected using Bonferroni adjustment. *means remained significant after Bonferroni correction (adjusted threshold $p<0.00238$).
